# Regulation of Lineage Specific DNA Hypomethylation in Mouse Trophectoderm

**DOI:** 10.1371/journal.pone.0068846

**Published:** 2013-06-25

**Authors:** Masaaki Oda, David Oxley, Wendy Dean, Wolf Reik

**Affiliations:** 1 Epigenetics Programme, the Babraham Institute, Cambridge, United Kingdom; 2 Mass Spectrometry, the Babraham Institute, Cambridge, United Kingdom; 3 Centre for Trophoblast Research, University of Cambridge, Cambridge, United Kingdom; Michigan State University, United States of America

## Abstract

**Background:**

DNA methylation is reprogrammed during early embryogenesis by active and passive mechanisms in advance of the first differentiation event producing the embryonic and extraembryonic lineage cells which contribute to the future embryo proper and to the placenta respectively. Embryonic lineage cells re-acquire a highly methylated genome dependent on the DNA methyltransferases (DNMTs) Dnmt3a and Dnmt3b that are required for *de novo* methylation. By contrast, extraembryonic lineage cells remain globally hypomethylated but the mechanisms that underlie this hypomethylation remain unknown.

**Methodology/Principal Findings:**

We have employed an inducible system that supports differentiation between these two lineages and recapitulates the DNA methylation asymmetry generated *in vivo*. We find that *in vitro* down-regulation of *Oct3/4* in ES cells recapitulates the decline in global DNA methylation associated with trophoblast. The *de novo* DNMTs Dnmt3a2 and Dnmt3b are down-regulated during trophoblast differentiation. Dnmt1, which is responsible for maintenance methylation, is expressed comparably in embryonic and trophoblast lineages, however importantly in trophoblast giant cells Dnmt1fails to be attracted to replication foci, thus allowing loss of DNA methylation while implicating a passive demethylation mechanism. Interestingly, Dnmt1 localization was restored by exogenous Np95/Uhrf1, a Dnmt1 chaperone required for Dnmt1-targeting to replication foci, yet DNA methylation levels remained low. Over-expression of *de novo* DNMTs also failed to increase DNA methylation in target sequences.

**Conclusions/Significance:**

We propose that induced trophoblast cells may have a mechanism to resist genome-wide increases of DNA methylation, thus reinforcing the genome-wide epigenetic distinctions between the embryonic and extraembryonic lineages in the mouse. This resistance may be based on transcription factors or on global differences in chromatin structure.

## Introduction

Epigenetic modifications are required to ensure the faithful inheritance of gene expression and genome organization in development. Histone modifications tend to confer shorter-term and more flexible regulation, for example temporal silencing of developmental genes, which are required for later developmental events [1,2]. On the other hand, DNA methylation can be more stable and contributes to the long-term stability of gene regulation, for example silencing of transposons and monoallelic expression of imprinted genes [3]. DNA methylation states can be stably inherited during mitosis and total levels of DNA methylation are not significantly different between the different types of somatic cells, however, genome-wide DNA methylation patterns are reprogrammed twice in development, during gametogenesis and early embryogenesis. It is considered that the demethylation step of the reprogramming process may aid the acquisition of pluripotency while the subsequent re-methylation step establishes unique DNA methylation patterns specific to a particular cell type or developmental stage. 

During the reprogramming process of early development, the first differentiation event prior to implantation gives rise to the two cell lineages, the embryonic and extraembryonic trophoblast lineage which contributes to the embryo proper and the extraembryonic tissue respectively including the placenta. It is now largely accepted that interactions between signaling events, transcription factor networks, and epigenetic regulation are involved in establishing these first two cell lineages. For example, the transcriptional regulator *Elf5* which is important for trophoblast cell fate is epigenetically silenced by DNA methylation in embryonic lineage cells [4,5]. ES cells deficient in DNA methylation can efficiently differentiate into the trophoblast lineage. The idea that the first lineages of the embryo differ in both quantitative and qualitative aspects of DNA methylation is not new. Early studies, employing DNA methylation sensitive restriction analysis and later immunofluorescence staining for 5-methylcytosine, revealed widespread differences suggesting a highly methylated epiblast lineage and a comparatively hypomethylated extraembryonic lineage [6–8]. These results have been confirmed more recently using quantitative genome-wide approaches in conjunction with next generation sequencing [9]. Collectively, these results identify DNA methylation differences associated with both genic and structurally important regions that influence function, first defining and then reinforcing lineage-specific distinctions.

The asymmetric regulation of DNA methylation between the early lineages cannot be fully explained simply on the basis of expression of the *de novo* DNMTs. The highly methylated genome in the embryo proper is established after implantation in a Dnmt3-dependent manner, with Dnmt3b the dominant contributor to *de novo* methylation [10,11]. This is supported by the report that Dnmt3b protein is detected more in embryonic lineage cells compared to the extraembryonic lineage [12]. However, Dnmt3 dependent-*de novo* methylation actually occurs in extraembryonic trophoblast cells as well as embryonic lineage cells at some regions such as at the pluripotency gene *Oct3/4* (also known as *Pou5f1*) [13].

In this study, we tried to understand the mechanism giving rise to the difference in global DNA methylation levels between embryonic and extraembryonic lineage. To address this question, we have employed an inducible system that results in the extinction of *Oct3/4* expression and thus allows ES cells to undergo a progressive transition towards trophoblast cells. This allowed us to trace the changes in both DNA methylation levels and expression of DNMTs. We found that the DNA methylation levels of some repetitive and retrotransposable elements decreased during trophoblast differentiation, but not embryonic differentiation in the absence of LIF. Dnmt1, the maintenance methyltransferase, was not found in association with replication foci in trophoblast cells in contrast to ES cells and differentiated embryonic cells where Dnmt1 accumulated at replication foci, The expression of the Dnmt1 chaperone Np95 was also reduced in trophoblast differentiation compared to embryonic differentiation. Contrary to expectation, overexpression of the family members involved in DNA methylation failed to restore the DNA methylation level during trophoblast differentiation. This may imply that the hypomethylated state is independent of the expression levels of DNMTs and that it is regulated in trophoblast cells autonomously and intrinsically.

## Results

### Asymmetric DNA methylation in the embryonic and extraembryonic lineages

To investigate the regulation of DNA methylation in embryonic and extraembryonic lineage differentiation, we used mouse ZHBTc4 ES cells as a model system, in which both of the endogenous *Oct3/4* alleles are disrupted and a doxycycline (Dox)-regulatable *Oct3/4* transgene is expressed to maintain self-renewal. Inducible depletion of *Oct3/4* initiates the transdifferentiation of ZHBTc4 ES cells to trophoblast cells (ZHBTc4+Dox cells) efficiently [14]. As a control for differentiation, embryonic lineage cells (ZHBTc4-Lif cells) are also induced from ZHBTc4 ES cells by removal of LIF from ES cell culture medium. Quantitative RT-PCR analysis confirmed expression of the expected marker genes for undifferentiated cells, embryonic and trophoblast cells in the E9.5 conceptus and during ZHBTc4 differentiation (Figure S1): the mesoderm marker gene (*T*) and the primitive ectoderm gene (*Fgf5*) were expressed in the embryo proper and ZHBTc4-Lif cells, and trophectoderm marker genes (*Cdx2*, *Rhox6*, *Elf5*) and TG cells marker (*Plate 1*) were highly expressed in trophoblast cells and ZHBTc4+Dox cells. It is worth noting that in keeping with its expression in differentiated epithelial cells, *Elf5* which is repressed in ES cells, was up-regulated upon differentiation mediated by withdrawal of LIF [15]. *Oct3/4* was highly expressed in ZHBTc4 ES cells. During ZHBTc4+Dox differentiation, *Oct3/4* and *Zfp42* expression was rapidly extinguished and *Cdx2* was transiently detected from day 2 after induction, in keeping with the transition from one lineage to the other. In contrast upon differentiation to ZHBTc4-Lif cells *Oct3/4* and *Zfp42* expression decreased gradually.

First we examined total cytosine methylation by mass-spectrometric analysis of DNA from ZHBTc4 ES cells, ZHBTc4+Dox cells and ZHBTc4-Lif cells in addition to the embryo proper and trophoblast cells of the E9.5 conceptus (Figure S2). The embryo proper had more methylcytosine than trophoblast cells (mC/C is 0.0326 ± 0.0008 in embryo proper and 0.0153 ± 0.0006 in trophoblast cells, P value across embryo proper and trophoblast cells is less than 0.0001), in agreement with previous work [9]. Interestingly, we found that a decrease in cytosine methylation occurred during ZHBTc4+Dox differentiation, but not ZHBTc4-Lif differentiation (mC/C is 0.0373 ± 0.0011 in ZHBTc4 ES, 0.0337 ± 0.0005 in ZHBTc4+Dox and 0.0395 ± 0.0015 in ZHBTc4-Lif, P value across ZHBTc4+Dox and –Lif is 0.01404) (Figure S2).

**Figure 1:**
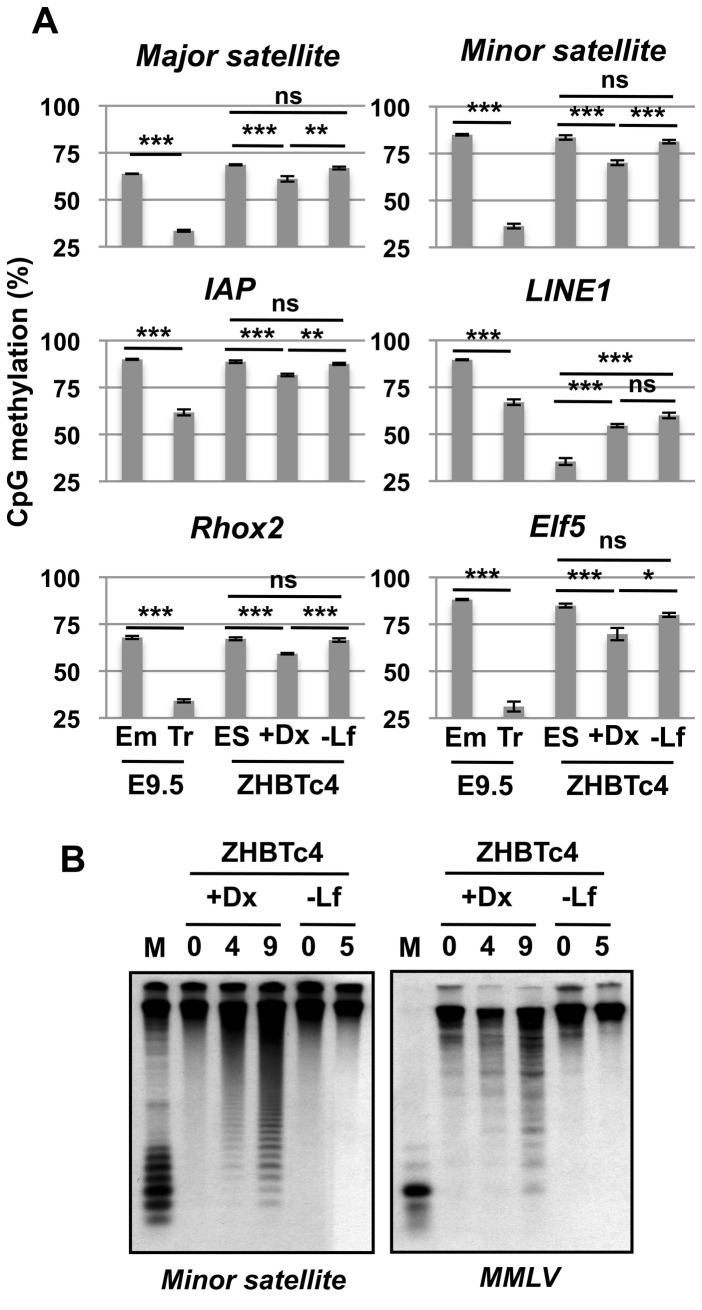
Asymmetric DNA methylation in the first two lineages. (A) The percentage of CpG methylation analyzed by Sequenom which averages the methylation of CpG methylation across each region. Embryo proper (Em) and trophoblast cells (Tr) are from E9.5 conceptus. ZHBTc4 ES-derived trophoblast cells (+Dx) and embryonic cells (-Lf) are differentiated by addition of doxycycline or removal of LIF respectively. Genomic DNA was collected at day 4 after differentiation. Values are means ± standard deviation (SD) of biological replicates (n=3-4). ***: p<0.001, **: *p*<0.01, *: *p*<0.05, ns: not significant; t-test and ANOVA followed by Tukey HSD post-hoc tests when appropriate. (B) DNA methylation of minor satellite and MMLV analyzed by southern blotting. Genomic DNA was collected from undifferentiated ES cells (0), trophoblast cells day 4 (4) or day 9 (9) after doxycycline treatment (+Dx), and embryonic cells day 5 (5) after the removal of LIF (-Lf). Genomic DNA was digested with methylation sensitive restriction enzyme HpaII and analyzed with each probe. Digestion with methylation-insensitive restriction enzyme MspI (M) is a control.

Lineage specific regulation of DNA methylation is tightly associated with specific sequences. High copy number elements in the genome including repetitive elements (centromeric minor satellite repeats and pericentromeric major satellite repeats) and interspersed retrotransposon repeats (IAP and LINE 1) are abundantly distributed in the mouse genome [16], and methylation levels of these elements tend to reflect the global methylation levels. Structurally relevant highly repeated centromeric satellites are hypermethylated in the embryo proper and hypomethylated in extraembryonic trophoblast cells at E9.5 (Figure 1A). Methylation of retrotransposons of the IAP and LINE 1 families also shows this pattern albeit to a lesser degree. Extraembryonic lineage-specific genes, *Rhox2* and *Elf5*, are also hypomethylated in trophoblast cells when compared to the embryo proper. DNA methylation analysis during *in vitro* ZHBTc4 differentiation conforms to the trend identified in E9.5 conceptus. ZHBTc4+Dox cells showed lower methylation at all analyzed regions when compared against ZHBTc4-Lif cells although these differences are small in comparison to those from *in vivo* tissues (22-52% lower in trophoblast cells than embryo proper and 6-11% lower in ZHBTc4+Dox cells than ZHBTc4-Lif cells). Interestingly a reduction in DNA methylation was evident upon differentiation of ZHBTc4 ES cells to ZHBTc4+Dox cells at all regions analyzed except for at LINE 1 elements where the methylation level is higher in ZHBTc4+Dox cells than in ZHBTc4 ES cells (Figure 1A). DNA methylation analysis by Southern blotting also confirmed the decrease of DNA methylation at minor satellites and MMLV during ZHBTc4+Dox but not ZHBTc4-Lif differentiation (Figure 1B).

An interesting departure from this general pattern is observed in LINE 1 sequences where relatively hypomethylated repeats are characteristic of ES cells [17] while differentiated cell types of either lineage become hypermethylated, showing that trophoblast lineage cells have the capacity for *de novo* methylation (Figure 1A). Some genes including the pluripotency gene *Oct3/4* are also known to be more methylated in trophoblast cells than ES cells [18,19]. Thus the methylation increase in LINE 1 elements may be largely attributed to differentiation status rather than cell type. Taken together, our data suggest that a general decrease in methylation is largely characteristic of trophoblast differentiation although variations from this rule occur in a sequence specific manner.

Total cytosine methylation levels in trophoblast stem (TS) cells are comparable to ES cells (Figure S3A). Interestingly, differentiation from TS cells to trophoblast giant (TG) cells also coincided with a decline in global DNA methylation. This suggests that further differentiation rather than commitment to trophectoderm lineage may be responsible for the observed decline in DNA methylation. DNA methylation levels at IAP and *Rhox2* decreased in a similar fashion to total methylation levels during the transition from TS cells to TG cells (Figure S3B). DNA methylation levels at minor satellites and major satellites were already lower in TS cells than ES cells, and further reduction was observed during the differentiation process from TS cells to TG cells. Methylation levels of LINE 1 elements were higher in TS cells than ES cells, but a reduction was observed in TG cells contrary to ZHBTc4 differentiation where an increase of methylation levels was seen. DNA methylation at *Elf5* was consistently low in TS cells and TG cells as reported previously [5]. 

### mRNA expression of DNA methyltransferases in the first two lineages

**Figure 2:**
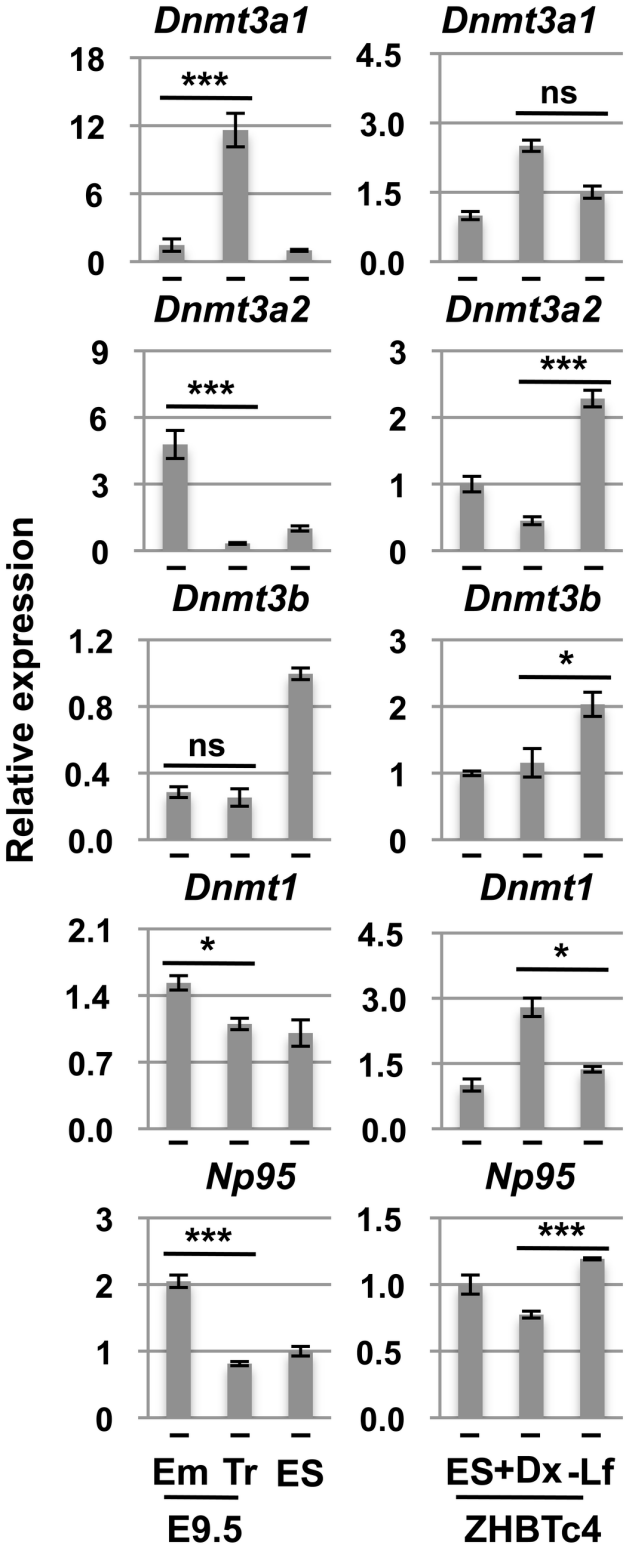
Differential expression of DNA methyltransferases in the first two lineages. mRNA expression of DNMTs and Np95 in E9.5 conceptus (Em: embryo proper, Tr: trophoblast cells), ZHBTc4 ES cells (ES), ZHBTc4-derived trophoblast cells (+Dx) and ZHBTc4-derived embryonic cells (-Lf). The value of ES cell is set to 1.0. Values are means ± SD of biological replicates (n=3-4). ***: *p*<0.001, **: *p*<0.01, *: *p*<0.05, ns: not significant; Mann-Whitney and ANOVA followed by Tukey HSD post-hoc tests when appropriate. The data for +Dx and –Lf were collected at day 2 for *Dnmt3a2* and at day 4 for *Dnmt3a1*, *Dnmt3b*, *Dnmt1* and *Np95*. Data for marker gene expression and the time course experiments are shown in supporting information (Figure S3).

Mechanistically one of the simplest explanations for the differences in DNA methylation would be a difference in expression levels of DNMT family members and their chaperones. The expression of *de novo* DNMTs (Dnmt3s) was unequal between embryonic cells and trophoblast cells. Both embryonic and trophoblast cells express Dnmt3a isoforms abundantly *in vivo*, however, a strict division of labour is in place making the short isoform *Dnmt3a2* enriched in the embryo proper and the long isoform, *Dnmt3a1*, similarly enriched in the trophoblast cells (Figure 2). These trends are widely maintained in the *in vitro* differentiation system (Figure 2). Dnmt3a1 and Dnmt3a2 are both enzymatically active, but these isoforms show differences in subcellular localization, expression pattern and interaction with Dnmt3L [20–25]. Unlike Dnmt3a1, which is concentrated in heterochromatin, Dnmt3a2 localizes to euchromatin [20]. Dnmt3L physically interacts with Dnmt3a2, but not Dnmt3a1 [25]. *Dnmt3b* was highly expressed in ZHBTc4 ES cells, however it appears that expression of both *Dnmt3b* and *Dnmt3a2* increased right after differentiation followed by a decrease (Figure S1B). A difference in *Dnmt1* expression was observed between ZHBTc4-Lif and ZHBTc4+Dox cells but in both cell types, expression was higher than in ZHBTc4 ES cells. *Dnmt1* expression increased by day 4 of ZHBTc4+Dox differentiation, but decreased after that. This change, however, does not correlate with the decline in DNA methylation level, implying that the expression level of *Dnmt1* might not relate to the decline in DNA methylation in extraembryonic cells as reported previously in human placenta [26].

From the expression data above, we hypothesized that lower expression of either of the Dnmt3s might cause the difference in DNA methylation between embryonic and extraembryonic lineage cells. The Dnmt3s are the main enzymes responsible for *de novo* DNA methylation [10], but they are also involved in the maintenance of genomic methylation patterns in mouse embryonic stem cells in certain sequence contexts [21].

### Overexpression of Dnmt3 family members does not inhibit the decline in DNA methylation during trophoblast differentiation

**Figure 3:**
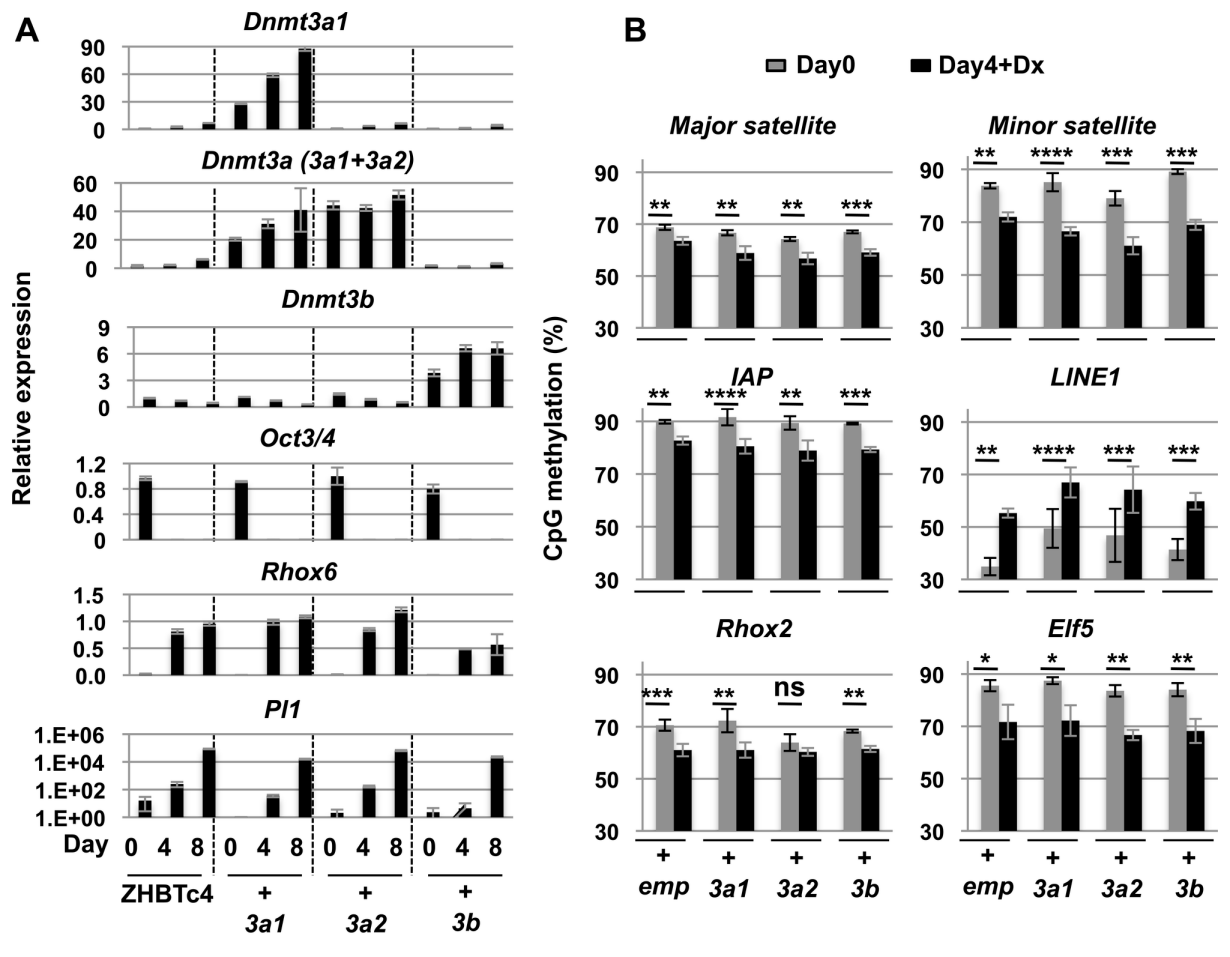
Overexpression of the Dnmt3 genes fail to restore somatic level of DNA methylation in trophoblast differentiation. (A) mRNA expression of *Dnmt3* genes, *Oct3/4*, *Rhox6* and Plate *1* during ZHBTc4 trophoblast differentiation with/without overexpression of exogenous Dnmt3a1 (3a1), Dnmt3a2 (3a2) or Dnmt3b (3b). A representative clone from each group of Dnmt3-expressing stable clone is shown in the figure. Values are means ± SD of technical replicates (n=3). Other clones in each group showed similar levels of expression of Dnmt3 genes and marker genes. (B) DNA methylation analysis by Sequenom in ZHBTc4 ES cells and trophoblast cells with/without overexpression of exogenous Dnmt3a1 (3a1), Dnmt3a2 (3a2) or Dnmt3b (3b). emp: empty vector. A gray column indicates ES cell data (Day 0). A black column indicates data from trophoblast cells induced by the addition of doxycycline (Day4+Dx). Values are means ± SD of biological replicates (n=3-5) except for the value of major satellite for empty vector day 4 whose value is shown as the mean of biological duplicate. So, there is no stats for the value of major satellite at day 4. ****: p<0.0001, ***: *p*<0.001, **: *p*<0.01, *: *p*<0.05, ns: not significant; paired t-tests.

To assess whether quantitative aspects of DNA methylation could be altered in cell lineages, stable integrations of the Dnmt3s were generated and the potential for forced overexpression of these enzymes on differentiation were evaluated (Figure 3). Overexpression of exogenous Dnmt3s was able to restore the DNA methylation in *Dnmt3a* ^*-/-*^ *Dnmt3b* ^*-/-*^ double knockout (DKO) ES cells (Figure S4A) [13], thus validating the activity of Dnmt3s transgenes. Although overexpression of Dnmt3s was expected to affect the trophoblast differentiation because high *de novo* activity was especially observed in embryonic cells around implantation, interestingly differentiation was not affected temporally and transdifferentiation between ES and TG cells proceeded as in the controls (Figure 3A).

Remarkably, despite providing functional enzymatic activities at significantly increased levels of expression (Figure 3A), DNA methylation at all targets remained unchanged upon differentiation with no differences compared to the control cell lines (Figure 3B). This suggests that Dnmt3s alone are not sufficient to increase methylation levels in trophoblast cells. This unexpected outcome led us to reconsider the involvement of Dnmt1 in global hypomethylation of trophoblast cells both *in vivo* and during induced transdifferentiation *in vitro*.

### Dnmt1 is excluded from replication foci in trophoblast giant cells

**Figure 4:**
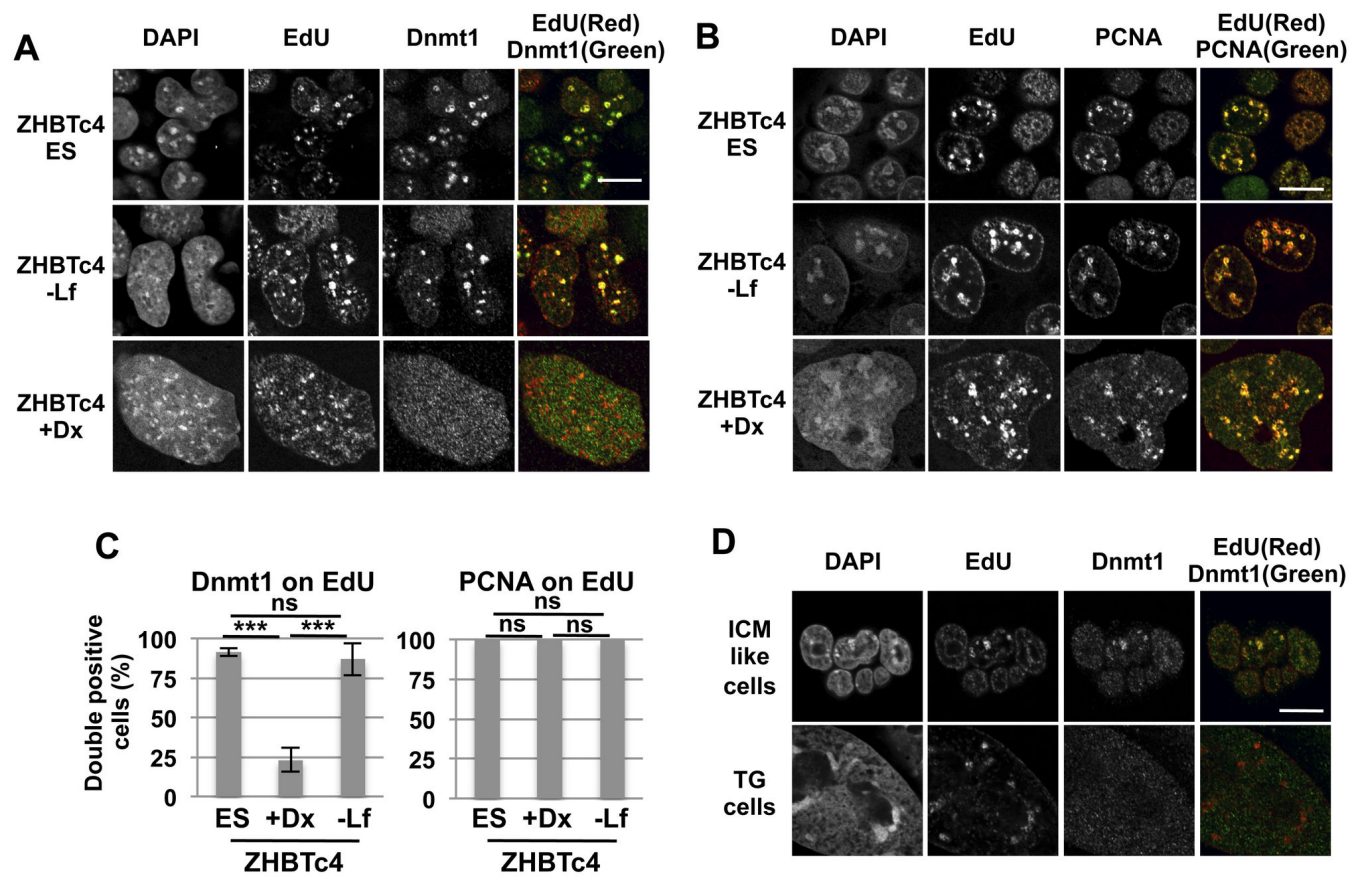
Lack of Dnmt1 accumulation on replication foci in trophoblast giant cells. (A,B) Immunostaining analysis of ZHBTc4 ES cells and ZHBTc4-derived embryonic (-Lf) and trophoblast cells (+Dx) at day 4 after differentiation using antibodies against Dnmt1 (A) and PCNA (B). Replication sites were visualized by the incorporation of the nucleotide analogue EdU. DNA was visualized with DAPI. Merged images represent overlays of immunofluorescence signal of Dnmt1 or PCNA (green) and EdU (red). (C) The score of Dnmt1 or PCNA localization at replication site in ZHBTc4 ES cells and trophoblast cells at day 4 after doxycycline induction (+Dx). Nuclear size was analyzed by ImageJ and divided into three categories (size similar to nucleus of ES cells, twice the size, and larger). Values are means ± SD of biological replicates (n=4-7 for Dnmt1, n=3-4 for PCNA). ***: p<0.001, ns: not significant; ANOVA and Bonferroni’s multiple comparison test. (D) Immunostaining analysis of blastocyst outgrowths using antibodies against Dnmt1. DNA and replication sites were visualized with DAPI and EdU respectively. Scale bar, 10 µm.

In its role as the primary maintenance methyltransferase, Dnmt1 ordinarily associates with replication foci in S phase in order to restore DNA methylation to the newly synthesized strand of the advancing replication fork at CpG dinucleotides [27]. According to the transcriptional data, the change of *Dnmt1* expression level did not match the decline in DNA methylation during ZHBTc4+Dox differentiation (Figure 2 and Figure S1). The abundance of Dnmt1 protein is regulated in a cell cycle dependent manner in that Dnmt1 is degraded outside of S-phase by proteasome regulation [28,29]. It is proposed that phosphorylation, methylation and ubiquitination could decrease the stability of Dnmt1. To focus on Dnmt1 protein in replicating cells during S-phase, we stained for Dnmt1 and visualized newly replicated DNA by pulse labeling with nucleotide analog, EdU. Pulse labeling of ZHBTc4 ES cells and ZHBTc4-Lif cells (-Lf) with EdU confirmed the co-localizing association of Dnmt1 with newly replicated foci (Figure 4A). The presence of Dnmt1 was confirmed in ZHBTc4+Dox cells (+Dx), however, enrichment of Dnmt1 was not observed on the replication foci.

Double scoring for Dnmt1 confirmed that cells undergoing ZHBTc4+Dox differentiation showed reduced association of Dnmt1 to replication foci (Figure 4C Table S1). ZHBTc4+Dox cells can possess enlarged or multiple nuclei [4]. Even ZHBTc4+Dox cells with small nuclei displayed loss of Dnmt1 at replication foci (Figure 4C). It appeared that Dnmt1 signals were detected in nuclei but did not accumulate at the replication foci. Dnmt1 localization in TS cells was the same as in ES cells, but TG cells induced from TS cells displayed similar localization patterns of Dnmt1 to that seen in ZHBTc4+Dox cells (Figure S3D). Antibody detection of proliferating cell nuclear antigen (PCNA) in close association with EdU foci confirmed both accessibility and methodology as appropriate (Figure 4B, C Table S1).

*In vivo* comparison of this pattern of association was confirmed by staining of Dnmt1 in EdU labeled blastocyst outgrowths where both embryonic and TG cells could be detected (Figure 4D). Taken together, the decline in DNA methylation was correlated with the lack of Dnmt1 localization during trophoblast differentiation. We speculate that there might be some regulation in the maintenance machinery involved in DNA replication during trophoblast differentiation.

### Dnmt1 chaperone, Np95/Uhrf1, is not detectable in nuclei of trophoblast giant cells

**Figure 5:**
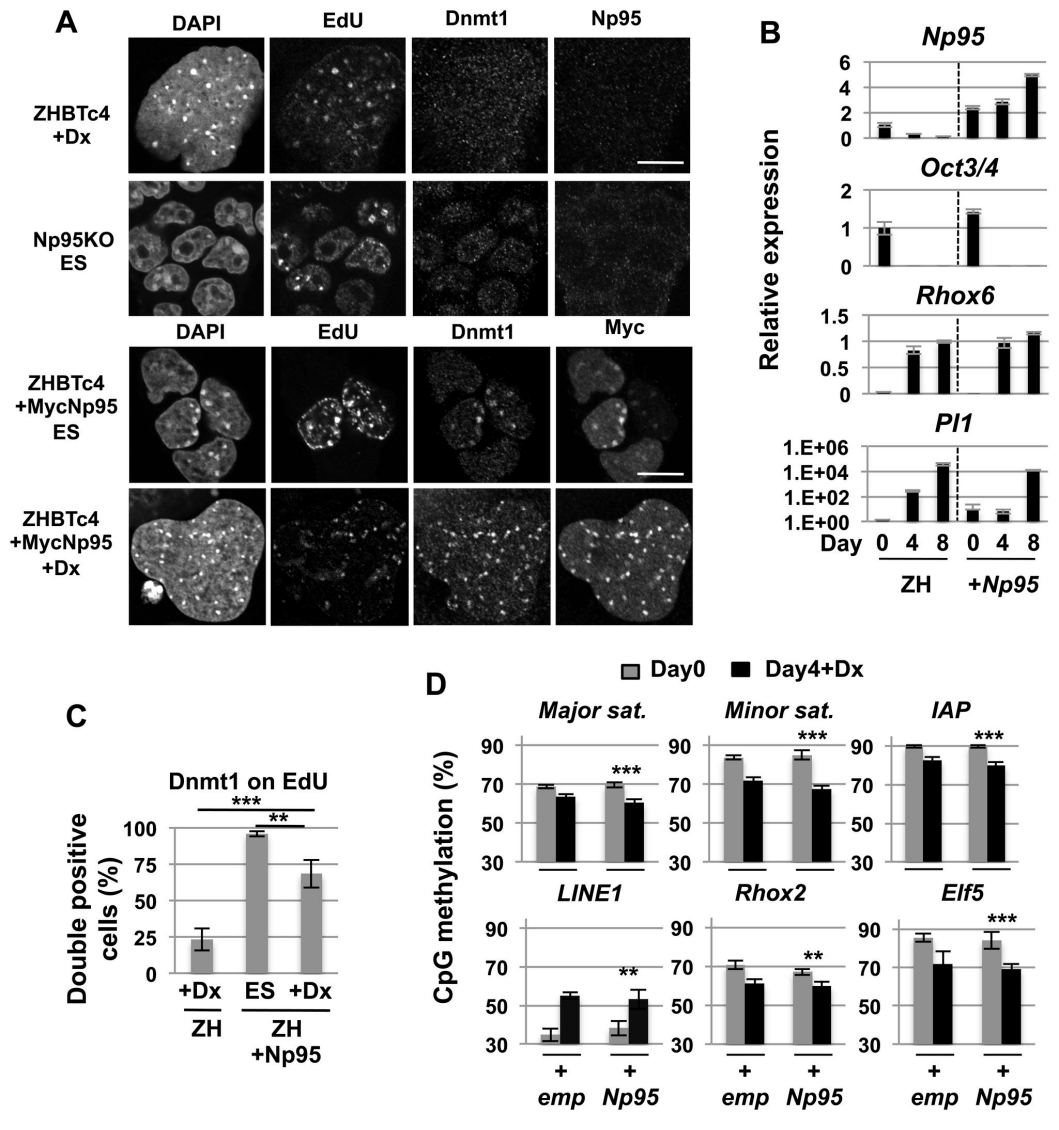
Overexpression of the Np95 gene fails to restore somatic levels of DNA methylation in trophoblast differentiation. (A) Immunostaining analysis of ZHBTc4-derived trophoblast cells (+Dx), Np95-KO ES cells, ZHBTc4 ES cells overexpressing exogenous Np95 (+MycNp95 ES) and ZHBTc4-derived trophoblast cells overexpressing exogenous Np95 (+MycNp95+Dx) using antibodies against Dnmt1, Np95 or Myc. DNA and replication sites were visualized with DAPI and EdU respectively. Scale bar, 10 µm. (B) mRNA expression of *Np95*, *Oct3/4*, *Rhox6* and Plate *1* genes during ZHBTc4 trophoblast differentiation with (+Np95) or without (ZH) overexpression of exogenous Np95. A representative clone for Np95-overexpressing ZHBTc4 cells is shown in the figure. Values are means ± SD of technical replicates (n=3). Other independent clones also showed similar results for marker gene and Np95 expression. (C) Dnmt1 localization at replication site in Np95-overexpressing ZHBTc4-derived trophoblast cells at day 4 after differentiation. The control data in ZHBTc4-derived trophoblast cells (ZH+Dx) is identical to ZHBTc4+Dx of Figure 4C. Values are means ± SD of biological replicates (n=3). ***: *p*<0.001, **: *p*<0.01, ns: not significant; ANOVA and Bonferroni’s multiple comparison test. (D) DNA methylation analysis by Sequenom in ZHBTc4 ES (Day0) and ZHBTc4-derived trophoblast cells (Day4+Dx) with (+Np95) or without (ZH) overexpression of exogenous Np95. The control data (+emp) is identical to the data of +emp in Figure 3B. Values are means ± SD of biological replicates (n=3-5). ***: *p*<0.001, **: *p*<0.01; paired t-tests.

Upon ZHBTc4+Dox differentiation Np95/Uhrf1, an obligate chaperone to Dnmt1, is down regulated over time (Figure 2B). To evaluate whether this could account for the intrinsic reduction in DNA methylation in trophectodermal derivatives, gain of function experiments were conducted with stable overexpression of Np95 (Figure 5). Although Dnmt1 is the main enzyme for maintenance methylation, it requires the chaperone Np95 for targeting to hemimethylated sites on the daughter strand at DNA replication foci during S-phase [30,31]. The transcription level of Np95 was lower in E9.5 trophoblast cells or ZHBTc4+Dox cells than embryo proper or ZHBTc4-Lif cells (Figure 2A and B). Furthermore, its expression decreased during the differentiation from TS cells to TG cells (Figure S3C). There is, however, still the possibility that the difference in transcriptional level can be caused by the difference of cell cycle status because the expression of Np95 is tightly regulated in a cell cycle dependent manner [32].

To focus on the Np95 protein itself in S-phase cells, we stained Np95 and visualized S-phase cells by EdU. As reported previously, Np95 localized to replication foci together with Dnmt1 in ES cells and TS cells (Figure S3D). However, we found that Np95 was not localized to replication foci of TG cells derived from both ZHBTc4 cells and TS cells (Figure 5A and Figure S3D). It appeared that Np95 was completely absent from nuclei of TG cells as seen in *Np95* ^*-/-*^ knockout (KO) ES cells. Next we hypothesized that repression of Np95 might result in mis-localization of Dnmt1 and/or result in a reduction of DNA methylation during trophoblast differentiation.

#### Exogenous Np95 improves Dnmt1 localization to replication foci but does not affect DNA methylation dynamics in trophoblast giant cells

On induced transdifferentiation, ZHBTc4+Dox cells have no detectable levels of Np95 (Figure 5A). To attempt to restore DNA methylation mediated by Dnmt1 recruitment, forced overexpression of a Myc-tagged Np95 (MycNp95) was engineered to be coupled to ZHBTc4 transdifferentiation (ZHBTc4+Dox+MycNp95).

It was confirmed that exogenous MycNp95 was targeted to replication foci and capable of restoring DNA methylation in *Np95* KO ES cells (Figure S4B, C) [30]. The *MycNp95* gene was more highly expressed than endogenous *Np95* gene and stably expressed even after ZHBTc4+Dox differentiation (Figure 5B). ZHBTc4+Dox+MycNp95 cells showed similar expression levels of Plate *1* and *Rhox6* and were morphologically similar to control ZHBTc4+Dox cells (Figure 5B and data not shown). Immunostaining confirmed the co-localization of MycNp95 and Dnmt1 to EdU labeled replication foci in middle-late S phase in ZHBTc4 ES+MycNp95 cells and in ZHBTc4+Dox+MycNp95 cells (Figure 5A and C). Thus overexpression of MycNp95 was able to improve Dnmt1 association to replication sites *in vitro*. Importantly temporal progression did not appear to be affected by the 5-fold increase in Np95 expression (Figure 5B). However, despite driving Dnmt1 to replication foci by overexpression of MycNp95, DNA methylation of target sequences was unaffected (Figure 5D). Thus, lower expression of Np95 may not account for global hypomethylation during trophoblast differentiation. 

Thus, decline in DNA methylation in ZHBTc4+Dox differentiation is not simply a consequence of the absence of Dnmt1 at replication forks owing to insufficient expression of an obligate chaperone, Np95. Rather loss of DNA methylation appears to be a consequence of some intrinsic setting of DNA methylation to ensure a hypomethylated landscape in the trophectoderm lineage.

## Discussion

In this study, using an *in vitro* differentiation system of ES and TS cells which can recapitulate the developmental processes around implantation, we focused on the first two cell lineages in development, embryonic and extraembryonic trophoblast cells, as a model system to address cell-type specific regulation of DNA methylation. *In vivo*, demethylation begins in the zygote and by the blastocyst stage (by which the trophectoderm has been established) global hypomethylation has been achieved [33], which is then largely maintained in the trophoblast lineage. This demethylation is achieved by a combination of active (including hydroxylation by the Tet family of enzymes) and passive processes (including cytoplasmic retention of Dnmt1). By contrast, there is dramatic *de novo* methylation in embryonic tissues starting at implantation which depends on Dnmt3a and Dnmt3b. Interestingly, DNA methylation deficient ES cells efficiently differentiate into extraembryonic cells [5].

Here we found that *in vitro* trophoblast differentiation qualitatively recapitulates this decrease in global DNA methylation levels while embryonic differentiation does not. This reflects the asymmetry of global DNA methylation levels in the two lineages *in vivo*; high methylation in the embryo proper and low methylation in extraembryonic trophoblast cells. Although *in vitro* trophoblast cells doesn’t fall to the methylation level of *in vivo* trophoblast cells, their difference in methylation (higher in ZHBTc4+Dox cells than E9.5 trophoblast cells) is likely due to their origin. E9.5 trophoblast cells are derived from the early blastocyst which has low levels of DNA methylation in early embryogenesis. On the other hand ZHBTc4+Dox cells are induced from ES cells whose methylation status is closer to that of the epiblast after *de novo* methylation. This decline in DNA methylation occurs at selective regions; we therefore investigated the mechanisms behind the decline. We found that *de novo* DNMTs, *Dnmt3a2* and *Dnmt3b* and the obligate Dnmt1 chaperone *Np95* were expressed at lower levels during ZHBTc4+Dox than in ZHBTc4-Lif differentiation. Since it is known that Dnmt3b, for example, is required for the maintenance methylation of minor satellites, a lower expression level of Dnmt3a and Dnmt3b could therefore contribute to the loss of methylation [21]. Their overexpression, however, did not impede the decline in DNA methylation level during ZHBTc4+Dox differentiation.

TS cells are already committed to the trophectoderm lineage and show lower levels of DNA methylation compared to ES cells at some regions including minor satellites, major satellites and at *Elf5*. Interestingly, however, an additional decline in DNA methylation was observed in completely methylated regions such as IAP elements and at *Rhox2* during the differentiation from TS cells to TG cells, suggesting that the same mechanism affecting the change in DNA methylation status might be in operation in both ZHBTc4+Dox and TS differentiation systems. Importantly, during ZHBTc4+Dox and TS differentiation, we found that Dnmt1 accumulation at replication foci during S phase was impaired. This was also observed in *in vivo* blastocyst derived-TG cells, but not ICM-like cells. Maintenance methylation by Dnmt1 is coupled with DNA replication during S phase. It appears that exogenous MycNp95 recruited Dnmt1 to the replication foci, however Dnmt1 and EdU negative foci in ZHBTc4+Dox+MycNp95 cells we also observed. Restoration of Dnmt1 localization by exogenous MycNp95 might not be dependent on hemimethylated DNA, but on other histone marks such as H3K9 methylation [34], although exogenous MycNp95 restored both Dnmt1 localization and DNA methylation level in *Np95* KO ES cells. Collectively, the mechanism responsible for global hypomethylation is likely a combination of reduced levels of the *de novo* methyltransferases, of *Np95*, and importantly the failure to target Dnmt1 to replication foci. Nonetheless, the loss of methylation observed in our system is not as dramatic as that occurring during preimplantation development, probably because key mechanistic components are different.

In addition to providing a detailed description of methylation dynamics during trophoblast differentiation *in vitro*, our study provides novel insights into a mechanism that apparently maintains the hypomethylated state once it has been successfully established. Hence, contrary to expectation exogenous expression of *Np95* or the *de novo* methyltransferases in our differentiation system was unable to restore DNA methylation. This is despite expression of Np95 restoring targeting of Dnmt1 to replication foci, and despite exogenous expression of Dnmt3a and Dnmt3b being able to restore methylation in ES cells deficient in the methyltransferases, an important control in our experiments. We therefore conclude that trophoblast cells appear to have an inherent resistance against increases in DNA methylation.

This protection could take a number of forms. First, DNA-binding factors might protect DNA from being accessed by DNMTs. This might be in analogy to a recent study which shows that transcription factor binding sites can protect transgenes from *de novo* methylation in ES cells [35]. Secondly, since specific histone modifications can either attract or repel the *de novo* methyltransferases [36,37], the global chromatin structure in trophoblast cells which is clearly different to that in ICM and ES cells [38–40] may have a generally inhibitory effect on *de novo* methylation. Thirdly, trophoblast cells may undergo continuous active demethylation, keeping them hypomethylated despite the exogenous expression of methyltransferases. This last explanation is perhaps less likely because we did not detect elevated expression of Tet hydroxylases during *in vitro* trophoblast differentiation (data not shown).

The insights into regulation of DNA hypomethylation in mouse trophectoderm obtained in our study may also be relevant to the acquisition or maintenance of hypomethylation in other lineages, including in primordial germ cells where the exclusion of Np95 is also suggested [41] and during differentiation in somatic cells, for example during erythropoiesis [42].

## Materials and Methods

### Mice and Cell Culture

All experimental procedures were conducted under licenses by the Home Office (UK) in accordance with the Animals (Scientific Procedures) Act 1986. Extraembryonic trophectoderm tissues and embryo proper from C57BL/6 were dissected under a microscope. Our quantitative RT-PCR analysis confirmed no contamination among extraembryonic tissue and embryo proper. Blastocyst outgrowths were obtained by culturing blastocysts from a cross of (C57BL/6 x CBA/Ca) F1 females mated to (C57BL/6 x CBA/Ca) F1 males in DMEM with 10% FBS for 3-5 days. 

ES cells were maintained as described previously [14]. The cells were grown on gelatin-coated culture dishes without feeder cells in standard ES cell medium.

For trophoblast differentiation by Oct3/4 down regulation, 1 µg/ml doxycyline (Sigma) was added to the ES culture medium of ZHBTc4 ES cells. For embryonic differentiation, LIF was withdrawn from ES culture medium. 

For replication labeling, cells were incubated for 10 min in medium containing 20 µM EdU (5-ethynyl-2’-deoxyuridine), a nucleotide analogue to thymidine, which is detected by click chemistry with the Click-iT kit (Invitrogen) and were harvested for immunostaining. 

Plasmid vectors for the expression of Dnmt3a, Dnmt3a2, Dnmt3b or Np95 were generated by subcloning the corresponding cDNAs into the pCAG-IRESpuro expression vector that contains the CAG promoter (a synthetic promoter that includes the chicken-β-actin promoter and the human cytomegalovirus immediate early enhancer) [13,30]. These constructs were individually electroporated into ZHBTc4 ES cells, which were subsequently selected in puromycin (Sigma) -containing medium for stable clones. 

### RNA expression analysis

Total RNA was isolated using an Allprep DNA/RNA mini kit (Qiagen) according to the manufacturers’ protocol. For RT-PCR, cDNA was synthesized from 0.5–1 µg of total RNA with random hexamers and Superscript III reverse transcriptase (Invitrogen). Quantitative PCR was performed with Brilliant II SYBR Green QPCR Master Mix (Agilent) using MX3005P (Stratagene) or CFX96 Real-Time system (Bio-Rad). *Hspcb* and *Atp5b* genes were used for normalization. Sequences of primers for PCR are available from the authors on request. 

### Immunostaining analysis

Cells cultured on coverslips were fixed with 4% paraformaldehyde (Sigma) for 15 min at room temperature or methanol (BDH) for 4 min at -20C, and permeabilised with PBS containing 0.5% Trixon X-100 for 20 min at room temperature. Fixed materials were blocked in 0.05% Tween-20 in PBS containing 1% BSA for 30 min at room temperature and incubated for 45 min at room temperature with primary antibodies against Dnmt1 (H-300, Santa Cruz), PCNA (PC10, Santa Cruz), Np95 (Th10), or Myc epitope tag (Millipore). Detection was achieved using Alexa Fluor 488, 568, 594 or 647 labeled anti anti-mouse, anti-rat or anti-rabbit IgG antibody (Invitrogen) as secondary antibodies. DNA was stained with 1 µg/ml DAPI (Invitrogen) and all samples were mounted in SlowFade Gold antifade reagent (Invitrogen). Images were acquired using a laser scanning confocal microscope (FV1000, Olympus). Nuclear sizes were measured using ImageJ software. 

### DNA methylation analysis

Genomic DNA was isolated using an Allprep DNA/RNA mini kit (Qiagen) according to the manufacture’s protocol. For methylation analysis of CpG units at specific regions using MassArray® system (Sequenom) analysis, Sodium bisulfite treatment of genomic DNA was performed using an Epitect Bisulfite Kit (Qiagen). Converted DNA was amplified by HotStarTaq DNA polymerase (Qiagen). Sequences of PCR primers and PCR conditions are available from the authors on request. The subfamily of LINE 1 analyzed in the Sequenom analysis is LINE 1 A.

For DNA methylation analysis by southern blotting, genomic DNA was digested with the CpG methylation-sensitive or –insensitive restriction enzymes HpaII (Fermentus) or MspI (Fermentus), and subjected to southern blotting. The blot was hybridized with probes for minor satellite repeats or C-type endogenous retroviruses (MMLV).

For the analysis of methylation within total cytosine by mass-spectrometry, genomic DNA was digested with DNA degradase plus (ZYMO RESEARCH) and subjected to mass spectrometry (liquid chromatography electrospray ionization tandem mass spectrometry). 

## Supporting Information

Figure S1 RT-qPCR analysis in E9.5 conceptus and ZHBTc4 cell differentiation. (A) mRNA expression in E9.5 conceptus (Em: embryo proper, Tr: trophoblast cells). Values are means ± SD of biological replicates (n=3-4) except for the value of Rhox2 for ES cells whose value is shown as the mean ± error bar of biological duplicate. (B) RNA expression during ZHBTc4 cell differentiation. Embryonic differentiation is shown as open square and dashed line (-Lf). Trophoblast differentiation is shown as closed circle and solid line (+Dx). Values are means ± SD of technical replicates (n=3).
(TIF)

Figure S2 Asymmetric DNA methylation in the first two lineages. Total amount of methylcytosine was analyzed by mass-spectrometry. Embryo proper (Em) and trophoblast tissue (Tr) are from E9.5 conceptus. ZHBTc4 ES-derived trophoblast cells (+Dx) and embryonic cells (-Lf) are differentiated by addition of doxycycline or removal of LIF respectively. The samples were collected at day 4 after differentiation. Values are means ± SD of biological replicates (n=3). ****: *p*<0.0001, **: *p*<0.01, *: *p*<0.05, ns: not significant; t-test and ANOVA followed by Tukey HSD post-hoc tests when appropriate.
(TIF)

Figure S3 Characterization of TS cells and TS-derived trophoblast cells. (A) Total amount of methylcytosine analyzed by mass-spectrometry in ES, TS, and TS-derived trophoblast giant (TG) cells. TG cells are day 5 after differentiation. Values are means ± SD of technical replicates (n=3). (B) DNA methylation analysis by Sequenom in ES, TS and TS-derived TG cells. TG cells are day 5 after differentiation. Values are means ± SD of technical replicates (n=3). (C) mRNA expression of *Np95*, *Dnmt1*, *Oct3/4*, *Zfp42*, *Cdx2*, Plate *1*, *beta-actin*, and *Gapdh* genes in wild-type ES, Np95^-/-^ KO ES, TS and TS-derived TG cells. PCR cycles are shown on the right. (D) Immunostaining analysis of ES, TS, and TS-derived TG cells using antibodies against Dnmt1 and Np95. Replication sites and DNA were visualized by the incorporation of nucleotide analogue EdU and DAPI respectively. Merged images represent overlays of immunofluorescence signal of Np95 (green) and Dnmt1 (red). Scale bar, 10 µm.
(TIF)

Figure S4 Exogenous expression of DNMTs or Np95 can restore DNA methylation in respective knock out ES cells. (A) DNA methylation profile of the genomic region around *Rhox6/9* (target of Dnmt3a and Dnmt3b), *Ube2a* (non-target of Dnmt3a and Dnmt3b) and the region without HpaII site (No HpaII) obtained by HpaII-digestion PCR. Genomic DNA was digested with (+) or without (-) HpaII which is sensitive to CG methylation and was subjected to PCR. Loss of *Rhox6/9* methylation was restored by the exogenous Dnmt3a1, Dnmt3a2 or Dnmt3b. Independent stable clones are shown as #1, #2 or #3. (B) The percentage of CpG methylation analyzed by Sequenom. Loss of methylation in *Np95* KO ES cells was restored by the exogenous Np95 (MycNp95). Values are means ± SD of technical replicates (n=3) except for the value of major satellite for Np95KO and +MycNp95 whose values are shown as the mean ± error bar of biological duplicate. (C) Immunostaining analysis of rescued (+MycNp95) *Np95* KO ES cells using antibodies against Dnmt1 and Myc which detects exogenous Np95. DNA and replication sites were visualized with DAPI and EdU respectively. Exogenous Np95 is properly enriched as expected on the replication foci in middle-late S phase cells which are overlapped with DAPI-dense region. Scale bar, 10 µm.
(TIF)

Table S1 Quantification of colocalization of Dnmt1 or PCNA on replication foci. The value of Peason’s r is shown in the table. Values are means ± SD of biological replicates (n=10). ****: p<0.0001, ns: not significant; t-test and ANOVA followed by Tukey HSD post-hoc tests when appropriate. To quantify colocalization event, images were analysed using the ImageJ colocalization plugin (Coloc_2). For the analysis, an area (3.96 x 3.96 µm) within each nucleus was selected that included replication foci on heterochromatic region (DAPI-dense region); the pixel intensity correlation of Dnmt1 and EdU (or PCNA and EdU) over this area was analyzed by Coloc_2.
(TIF)
