# Supplementary figures and images for: Regulation of Lineage Specific DNA Hypomethylation in Mouse Trophectoderm
Source: PLoS One. 2013 Jun 25;8(6):e68846. doi: 10.1371/journal.pone.0068846 (PMC3692478; doi:10.1371/journal.pone.0068846)

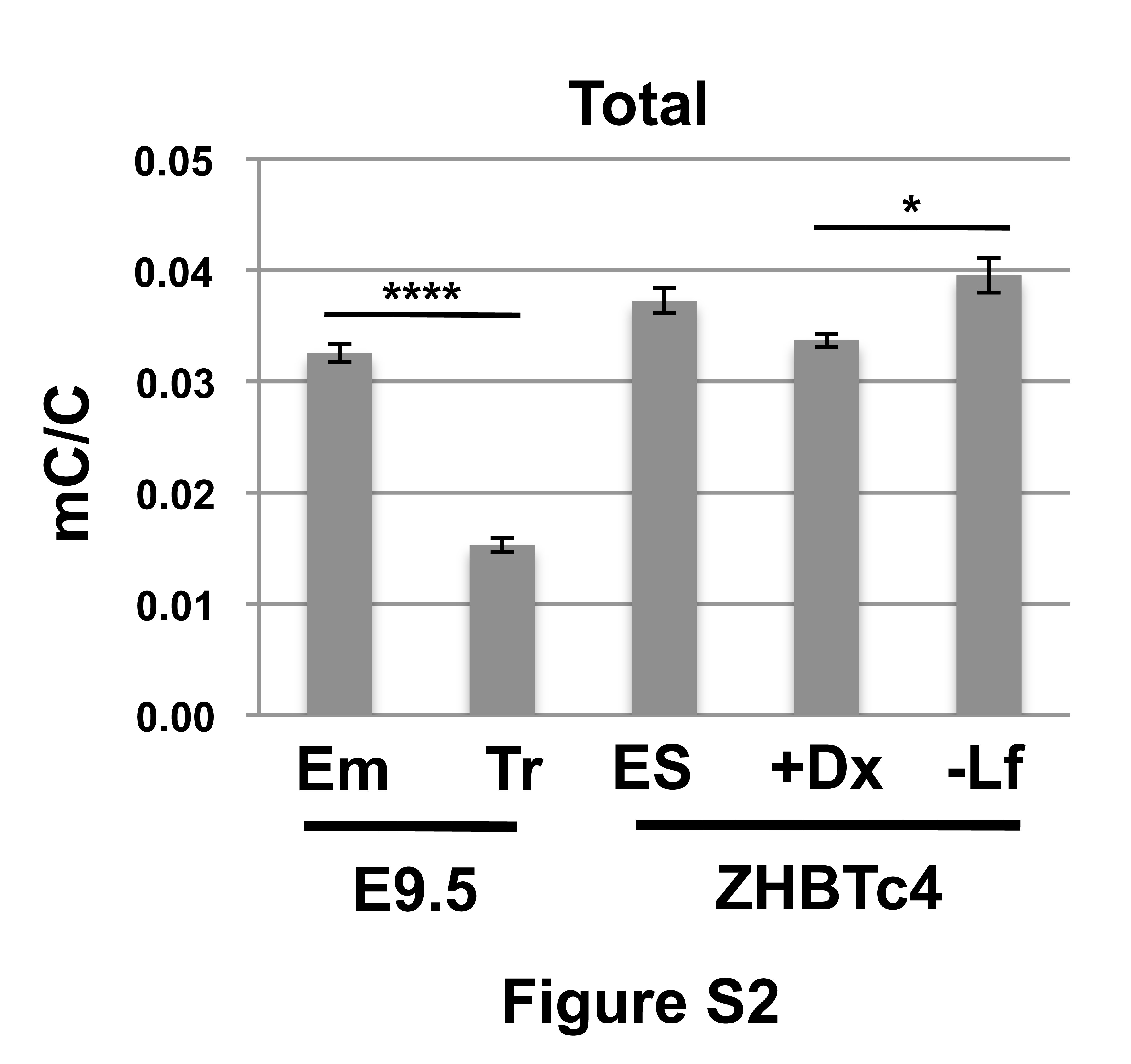

Supplement: Figure S2 — Total amount of methylcytosine was analyzed by mass-spectrometry. Embryo proper (Em) and trophoblast tissue (Tr) are from E9.5 conceptus. ZHBTc4 ES-derived trophoblast cells (+Dx) and embryonic cells (-Lf) are differentiated by addition of doxycycline or removal of LIF respectively. The samples were collected at day 4 after differentiation. Values are means ± SD of biological replicates (n=3). ****: p<0.0001, **: p<0.01, *: p<0.05, ns: not significant; t-test and ANOVA followed by Tukey HSD post-hoc tests when appropriate. (TIF) [file pone.0068846.s002.tif]

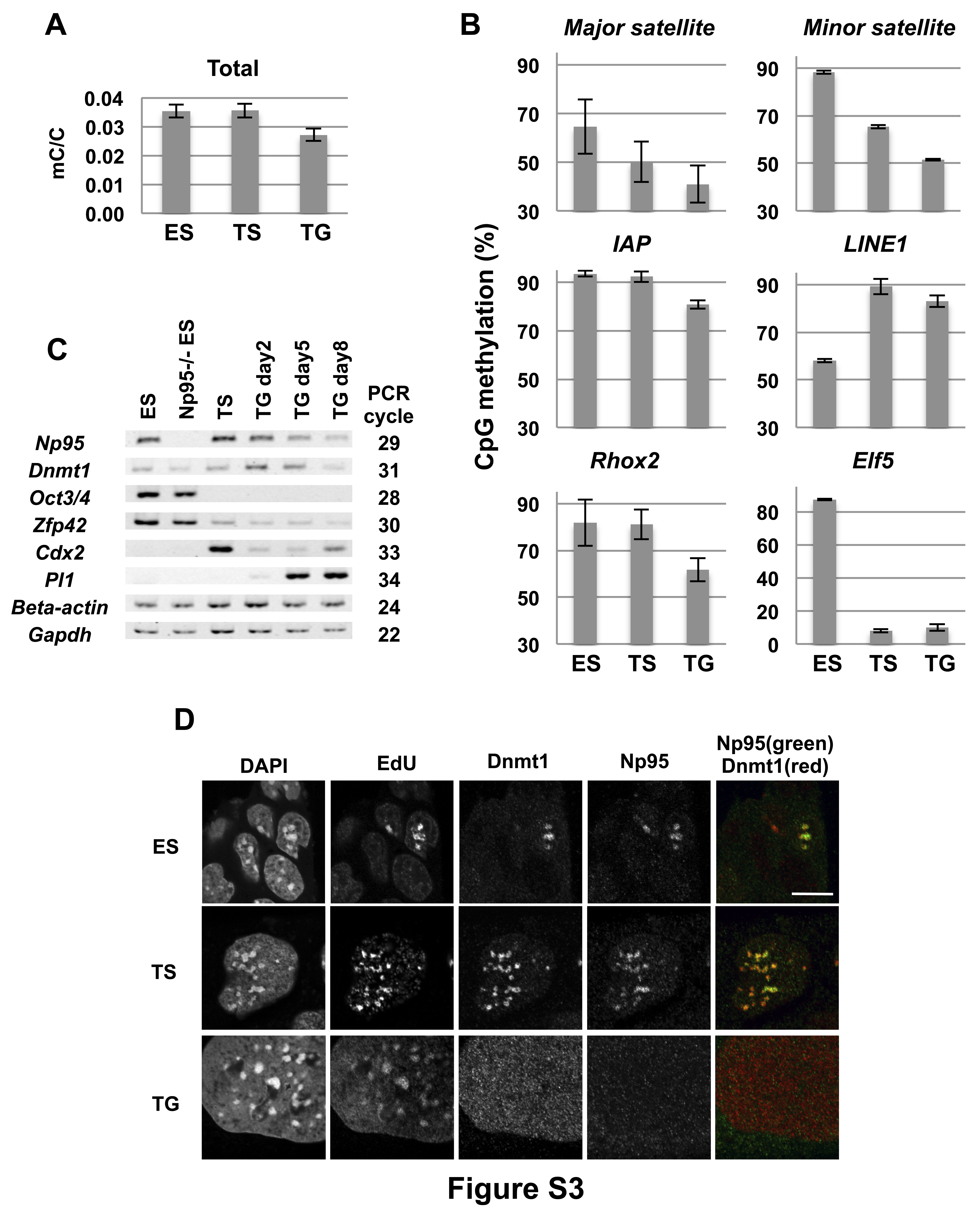

Supplement: Figure S3 — (A) Total amount of methylcytosine analyzed by mass-spectrometry in ES, TS, and TS-derived trophoblast giant (TG) cells. TG cells are day 5 after differentiation. Values are means ± SD of technical replicates (n=3). (B) DNA methylation analysis by Sequenom in ES, TS and TS-derived TG cells. TG cells are day 5 after differentiation. Values are means ± SD of technical replicates (n=3). (C) mRNA expression of Np95, Dnmt1, Oct3/4, Zfp42, Cdx2, Plate 1, beta-actin, and Gapdh genes in wild-type ES, Np95-/- KO ES, TS and TS-derived TG cells. PCR cycles are shown on the right. (D) Immunostaining analysis of ES, TS, and TS-derived TG cells using antibodies against Dnmt1 and Np95. Replication sites and DNA were visualized by the incorporation of nucleotide analogue EdU and DAPI respectively. Merged images represent overlays of immunofluorescence signal of Np95 (green) and Dnmt1 (red). Scale bar, 10 µm. (TIF) [file pone.0068846.s003.tif]

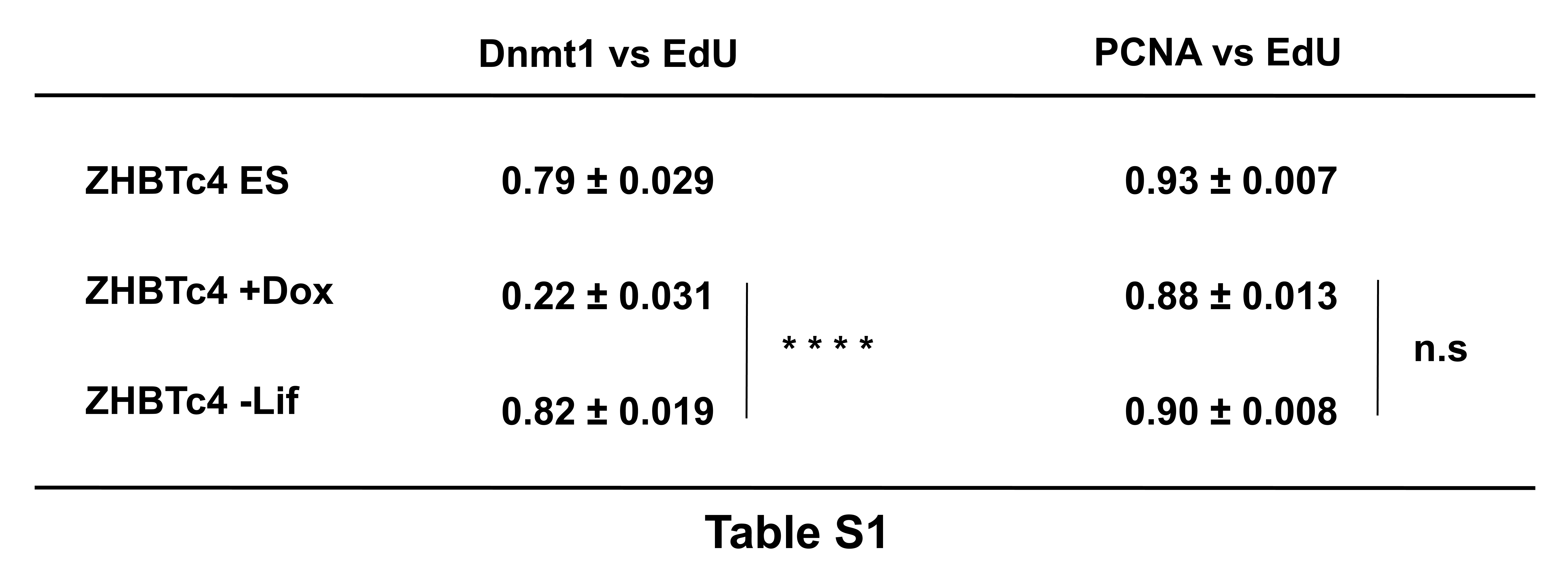

Supplement: Table S1 — The value of Peason’s r is shown in the table. Values are means ± SD of biological replicates (n=10). ****: p<0.0001, ns: not significant; t-test and ANOVA followed by Tukey HSD post-hoc tests when appropriate. To quantify colocalization event, images were analysed using the ImageJ colocalization plugin (Coloc_2). For the analysis, an area (3.96 x 3.96 µm) within each nucleus was selected that included replication foci on heterochromatic region (DAPI-dense region); the pixel intensity correlation of Dnmt1 and EdU (or PCNA and EdU) over this area was analyzed by Coloc_2. (TIF) [file pone.0068846.s005.tif]
